# Correction: Effectiveness and satisfaction of mindfulness-based cognitive therapy for children on anxiety, depression, and internet addiction in adolescents: Study protocol for a randomized control trial

**DOI:** 10.1371/journal.pone.0331382

**Published:** 2025-09-02

**Authors:** The PLOS One Staff

The first two authors, Masume Bakhtiari and Mojtaba Habibi Asgarabad, should be noted as contributing equally to the manuscript.

The publisher apologizes for the error.
